# Notch Signaling Regulates the Lifespan of Vascular Endothelial Cells via a p16-Dependent Pathway

**DOI:** 10.1371/journal.pone.0100359

**Published:** 2014-06-20

**Authors:** Yohko Yoshida, Yuka Hayashi, Masayoshi Suda, Kaoru Tateno, Sho Okada, Junji Moriya, Masataka Yokoyama, Aika Nojima, Masakatsu Yamashita, Yoshio Kobayashi, Ippei Shimizu, Tohru Minamino

**Affiliations:** 1 Department of Cardiovascular Biology and Medicine, Niigata University Graduate School of Medical and Dental Sciences, Niigata, Japan; 2 Department of Cardiovascular Medicine, Chiba University Graduate School of Medicine, Chiba, Japan; 3 Kazusa DNA Research Institute, Kisarazu, Chiba, Japan; 4 PRESTO, Japan Science and Technology Agency, Kawaguchi, Saitama, Japan; Osaka University Graduate School of Medicine, Japan

## Abstract

Evolutionarily conserved Notch signaling controls cell fate determination and differentiation during development, and is also essential for neovascularization in adults. Although recent studies suggest that the Notch pathway is associated with age-related conditions, it remains unclear whether Notch signaling is involved in vascular aging. Here we show that Notch signaling has a crucial role in endothelial cell senescence. Inhibition of Notch signaling in human endothelial cells induced premature senescence via a p16-dependent pathway. Conversely, over-expression of Notch1 or Jagged1 prolonged the replicative lifespan of endothelial cells. Notch1 positively regulated the expression of inhibitor of DNA binding 1 (Id1) and MAP kinase phosphatase 1 (MKP1), while MKP1 further up-regulated Id1 expression by inhibiting p38MAPK-induced protein degradation. Over-expression of Id1 down-regulated p16 expression, thereby inhibiting premature senescence of Notch1-deleted endothelial cells. These findings indicate that Notch1 signaling has a role in the regulation of endothelial cell senescence via a p16-dependent pathway and suggest that activation of Notch1 could be a new therapeutic target for treating age-associated vascular diseases.

## Introduction

The Notch pathway is a highly conserved signaling system that controls the fate and differentiation of cells during the development of various tissues. In mammals, the Notch signaling pathway is composed of four Notch receptors (Notch1 through 4) and five ligands (Jagged 1 and 2, and Delta-like 1, 3, and 4). All of the receptors and ligands are transmembrane proteins, so Notch signaling is often mediated by cell-cell interaction. Receptor-ligand interactions induce additional proteolytic cleavage, which frees the Notch intracellular domain (NICD) from the cell membrane. The NICD then translocates to the nucleus, where it associates with the DNA-binding protein CSL (Epstein-Barr virus latency C promoter binding factor 1 (CBF1; also known as RBPJ) in vertebrates, Suppressor of Hairless in Drosophila, and Lag1 in *C elegans*), displacing a histone deacetylase-co-repressor complex from CSL protein, so that transcription of Notch target genes is activated [1], [2]. In the cardiovascular system, Notch signaling has been implicated in the regulation of cardiomyocyte differentiation, the epithelial-to-mesenchymal transition during heart valve development, and vascular development. Therefore, mutations of Notch receptors or ligands cause congenital cardiovascular disorders such as Alagille syndrome, cerebral autosomal dominant arteriopathy with subcortical infarcts and leukoencephalopathy (CADASIL), and bicuspid aortic valve [3]–[5]. In adults, Notch signaling is essential for neovascularization and has been reported to be involved in age-associated conditions such as cancer, neurodegenerative disorders, and impaired regeneration of aged skeletal muscle [6]–[10].

Vascular cells have a finite lifespan in vitro and eventually enter a state of irreversible growth arrest called cellular senescence that is associated with various morphological changes and increased expression of senescence-associated molecules such as p53 or p16 [11], [12]. Accumulation of senescent vascular cells occurs in aged vessels, leading to an increase of inflammation combined with a decline of regenerative potential that promote vascular dysfunction and atherosclerosis [13]. Given that Notch signaling is involved in a wide range of pathophysiological processes, including age-associated conditions, we have examined the role of the Notch pathway in vascular aging.

Here we show that Notch signaling has a crucial role in endothelial cell senescence. Mechanistically, activation of Notch signaling up-regulates inhibitor of DNA binding 1 (Id1) and MAPK phosphatase 1 (MKP1) expression and prolongs endothelial cell lifespan by inhibiting a p16-dependent pathway. We further demonstrated that Notch1-induced up-regulation of MKP1 stabilizes Id1 protein by inhibiting p38MAPK-induced degradation, leading to prolongation of the endothelial cell lifespan. Moreover, overexpression of Id1 significantly attenuated p16 expression and increased the proliferative activity of endothelial cells in *Notch1*-deficient mice. Taken together, our results suggest that activation of Notch1 could be a new therapeutic target for treating age-associated vascular diseases.

## Materials and Methods

### Cell culture and reagents

Human umbilical vein endothelial cells (HUVEC) were purchased from Lonza (Walkersville, MD, USA) and cultured according to the manufacturer's instructions. Endothelial cell proliferation was assessed by counting cell numbers after subculture. We defined senescent cells as those that did not increase in number and remained subconfluent after 2 weeks of culture. Senescence-associated β-galactosidase (SA-β-gal) staining was performed as described previously [14]. The number of population doublings (PD) was calculated as follows: PD = log (number of cells obtained/initial number of cells)/log 2. In some experiments, endothelial cells were treated with SB203580 (WAKO, Osaka, Japan, 10 µM), anisomycin (WAKO, 2 µM) or MG132 (WAKO, 0.5 µM).

### Retroviral and lentiviral infection

The following plasmids were used for generating retroviral and lentiviral vectors: pLNCX or pLPCX (Clontech, Palo Alto, CA, USA) for a retroviral vector and pLVSIN (Takara, Tokyo, Japan) for a lentiviral vector. We created pLNCX-based vectors expressing full length Notch1, Notch1 intracellular domain (NICD), or Jagged1, and a pLPCX-based vector expressing inhibitor of DNA binding 1 (ID1), and a pLVSIN-based vector expressing inhibitor of DNA binding 1 (ID1). For knockdown experiments, we used the Knockout RNAi System (Clontech) or the MISSION lentiviral packaging system (Sigma, St. Louis, MO, USA), and generated retroviral or lentiviral vectors expressing short hairpin RNA (shRNA) targeting human Notch1, Jagged1, and INK4A according to the manufacturer's instruction. We used pLNCX, pLPCX, pSIREN-RetroQ, pLVSIN or pLKO.1 as negative control vectors. Human endothelial cells (passages 4–6) were plated at 5×10^5^ cells in 100-mm diameter dishes at 24 hours before infection. Then the culture medium was replaced by retroviral or lentiviral stock supplemented with 8 µg/ml polybrene (Sigma). From 48 hours after infection, cells were selected by culture in 500 µg/ml G418 (pLNCX-based vectors) or 0.5 µg/ml puromycin (pLPCX, pSIREN-RetroQ or pLKO.1-based vectors) for 7 days. After selection, 2×10^5^ cells were seeded in 100-mm diameter dishes on the 8th day post-infection, which was designated as day 0. For double infection, endothelial cells were infected with pLNCX-based vectors, purified with 500 µg/ml G418 for 7 days, and then subjected to infection with the second vector as described above.

### Animal models

All animal study protocols were approved by the Chiba University and Niigata University review boards. C57BL/6NCr mice were purchased from SLC Japan (Shizuoka, Japan) and Tie2-cre mice were obtained from Jackson Laboratories (Bar Harbor, ME, USA). Floxed Notch-1 mice have been described elsewhere [15]. Deletion of Notch1 in endothelial cells (EC) was accomplished by crossing male Tie2-cre^+^ mice with female *Notch1* ^lox/lox^ mice (Tie2-cre^+^; *Notch1* ^lox/+^, N1KO), and the corresponding littermates without the Cre transgene (Tie2-cre^−^; *Notch1* ^lox/+^) served as controls.

### Aortic ring assay

An *ex vivo* angiogenesis assay was performed as described previously[16] with slight modifications. Briefly, descending thoracic aortas from 8- to 10-week-old N1KO mice and littermate control mice were harvested and placed in Opti-MEM (GIBCO, Tokyo, Japan). The adventitia was dissected away and each aorta was cut into 1-mm rings under a dissecting microscope, which were cultured in Opti-MEM with lentivirus stock for 24 hours to introduce Id1 or Mock. Then the aortic rings were embedded in type I collagen gel in a 96-well plate supplemented with EGM-2 (Lonza) and 20 ng/ml VEGF, and cultured at 37°C. On day 7, cultured aortic rings were fixed with 4% formalin and stained with BS1 lectin-FITC (Sigma). Quantitative analysis of endothelial sprouting was performed by using images obtained with a Biorevo (Keyence Co., Osaka, Japan).

### Western blot analysis

Lysates were resolved by SDS-polyacrylamide gel electrophoresis. Proteins were transferred to a polyvinylidene difluoride membrane (Millipore, Bedford, MA, USA), which was incubated with the primary antibody followed by incubation with anti-rabbit, anti-mouse, or anti-goat immunoglobulin-G conjugated with horseradish peroxidase (Jackson ImmunoResearch, West Grove, PA, USA). Specific proteins were detected by using enhanced chemiluminescence (GE Healthcare, Backinghamshire, UK). The primary antibodies for Western blotting were as follows: anti-Notch1 antibody (Santa Cruz, Dallas, TX, USA), anti-Jagged1 antibody (Santa Cruz), anti-p53 antibody (DO-1) (Santa Cruz), anti-p21 antibody (Millipore, Billerica, MA, USA), anti-p16 antibody (BD Pharmingen, San Jose, CA, USA), anti-ID1 antibody (Santa Cruz), anti-phospho p38MAPK (Thr180/Tyr182) antibody (Cell signaling, Boston, MA, USA), anti-p38MAPK antibody (Cell signaling), anti-phospho SAPK/JNK (Thr183/Tyr185) antibody (Cell Signaling), anti-JNK1/3 antibody (Santa Cruz), anti-actin antibody (Cell signaling), anti-GAPDH antibody (Santa Cruz), anti-phosphoserine antibody (Abcam, Cambridge, UK) and anti-phosphothreonine antibody (Cell signaling). To assess the phosphorylation level of Id1, cell lysates were immunoprecipitated with FLAG M2 agarose (Sigma).

### RNA analysis

Total RNA (1 µg) was isolated from endothelial cells with RNA-Bee (TEL-TEST INC, Freindswood, TX, USA). Real-time PCR was performed by using a Light Cycler 480 (Roche, Basel, Swiss) with the Universal Probe Library and the Light Cycler 480 Probes Master (Roche) according to the manufacturer's instruction.

### DNA microarray analysis

HUVEC were infected with retroviral vectors encoding Jagged1, Jagged1-shRNA or Notch1-shRNA, or empty vector as control. Total RNA of them were isolated from HUVEC with RNA-Bee (TEL-TEST INC). Cyanine-3 (Cy3) labeled cRNA was prepared from 0.5 ug RNA using the One-Color Low RNA Input Linear Amplification PLUS kit (Agilent, Santa Clara, CA, USA) according to the manufacturer's instructions, and the resulting probes were hybridized to Agilent Whole Human Genome Oligo Microarrays (G4112F). The scanned images were normalized by Agilent GeneSpring GX software and differentially expressed genes were identified via the fold-change (FC) and p values of the *t*-test. Gene expression data is available through the Gene Expression Omnibus database (GSE40403).

### Measurement of telomere length

Telomere length was measured as described previously [17]. Briefly, genomic DNA was extracted from endothelial cells and telomeres were measured by real-time PCR using a Light Cycler 480 (Roche) with the LightCycler FastStart DNA Master SYBR Green kit (Roche) according to the manufacturer's instruction. The single-copy gene 36B4 (which encodes acidic ribosomal phosphor-protein) was used as the internal control.

### Chromatin immunoprecipitation assay

Chromatin immunoprecipitation (ChIP) was performed using chromatin prepared from Flag-tagged Notch1 over-expressing human endothelial cells or control cells (Mock). Sonicated chromatin was immunoprecipitated with antibodies targeting FLAG M2 (Sigma Aldrich), or normal mouse immunoglobulin G (IgG; Sigma Aldrich), and the precipitates were collected on protein A/G Sepharose beads (GE Healthcare). Real-time PCR was performed with the following primer pairs for CBF1 (forward: 5′-ttaattgatgatgtctctctcttttga-3′; reverse: 5′-tcaggaaaccaggaaaacca-3′) and beta-globin (forward: 5′-tgcaggctgcctatcagaa-3′; reverse: 5′-gcgagcttagtgatacttgtgg-3′).

### Statistical analysis

Data are shown as the mean ± SEM. Differences between groups were examined by Student's *t*-test or ANOVA followed by Bonferroni's correction for comparison of means. For all analyses, *P*<0.05 was considered statistically significant.

## Results

### Over-expression of Notch1 prolongs the lifespan of vascular endothelial cells

**Figure 1:**
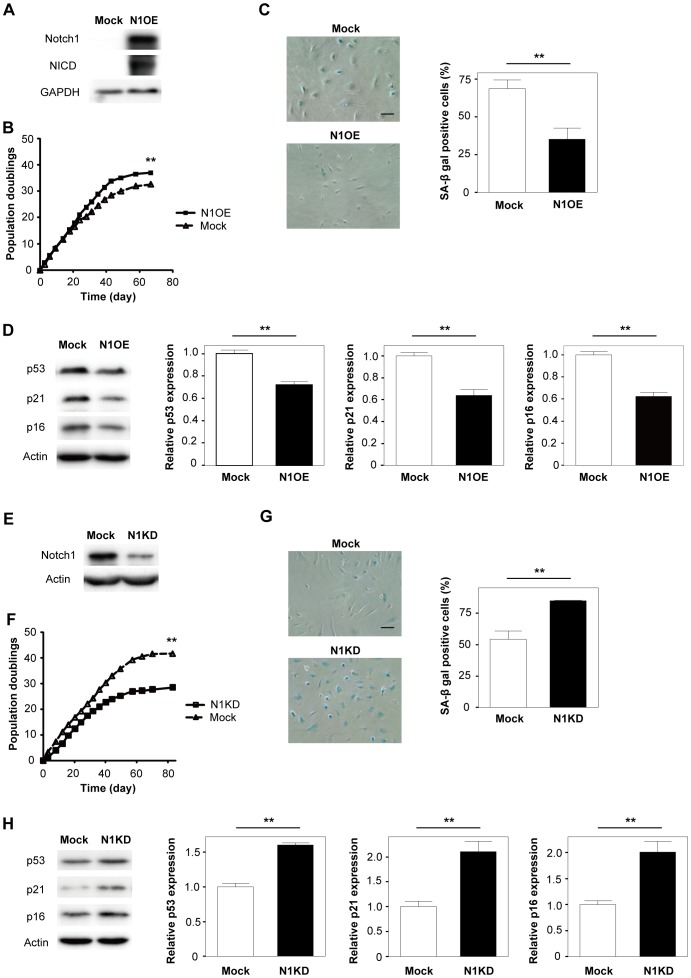
Over-expression of Notch1 prolongs the lifespan of vascular endothelial cells. (A) Western blot analysis of full length Notch1 (Notch1) and Notch intracellular domain (NICD) expression in human endothelial cells infected with a retroviral vector expressing Notch1 (N1OE) or the empty vector (Mock). GAPDH was used as the loading control. (B) Infected cells were passaged until senescence, and the total number of population doublings was determined (n = 3). (C) Senescence-associated β-galactosidase (SA-β gal) staining of endothelial cells prepared as in Figure 1A. Scale bar = 50 µm. The right graph shows quantitative data on SA-β gal-positive cells (n = 4). (D) Western blot analysis of p53, p21, and p16 expression in endothelial cells prepared as in Figure 1A. The right graph shows quantitative data on p53, p21, and p16 expression (n = 3). (E) Western blot analysis of full length Notch1 (Notch1) expression in human endothelial cells infected with a retroviral vector expressing Notch1 shRNA (N1KD) or sh-Control (Mock). (F) Infected cells were passaged until senescence, and the total number of population doublings was determined (n = 3). (G) Senescence-associated β-galactosidase (SA-β gal) staining of endothelial cells prepared as Figure 1E. Scale bar = 50 µm. The right graph shows quantitative data on SA-β gal positive cells (n = 4). (H) Western blot analysis of p53, p21, and p16 expression in endothelial cells prepared as in Figure 1E. The right graph shows quantitative data on p53, p21, and p16 expression (n = 3). All values represent the mean ± s.e.m. *P<0.05, **P<0.01.

To examine the role of the Notch pathway in endothelial senescence, we infected human endothelial cells with a retroviral vector encoding *NOTCH1* cDNA or an empty vector. Western blot analysis revealed that introduction of this construct led to stable up-regulation of Notch1 and its activation, as shown by up-regulation of Notch intracellular domain (NICD) (Figure 1A and Figure S1A). We examined the replicative lifespan of infected cells and found that up-regulation of Notch1 prolonged the lifespan of endothelial cells along with a decrease of senescence-associated β-galactosidase (SA-β-gal) activity and decreased expression of senescence-associated molecules such as p53, p21, and p16 (Figure 1B–D). We next examined the effect of Notch1 deletion on the lifespan of endothelial cells by using a retroviral vector encoding short hairpin RNA for Notch1. Disruption of Notch1 markedly reduced the maximum number of population doublings together with an increase of SA-β-gal activity and up-regulation of the expression of p53, p21, and p16 (Figure 1E–H). One widely discussed hypothesis of cellular senescence is the telomere hypothesis. Telomerase activity declines with aging because of a decrease in telomerase catalytic component (TERT) expression, leading to telomere shortening and cellular senescence. We examined telomere length and TERT expression and found that over-expression of Notch1 did not affect either of these factors (Figure S2A, B), suggesting that Notch signaling regulates vascular aging via a telomere-independent mechanism. We also found that introduction of NICD led to premature senescence of human endothelial cells along with up-regulation of negative regulators of cell cycle (Figure S1A-C), suggesting that constitutive activation of the Notch pathway negatively regulates cell lifespan.

### Over-expression of Jagged1 prolongs the lifespan of vascular endothelial cells

**Figure 2:**
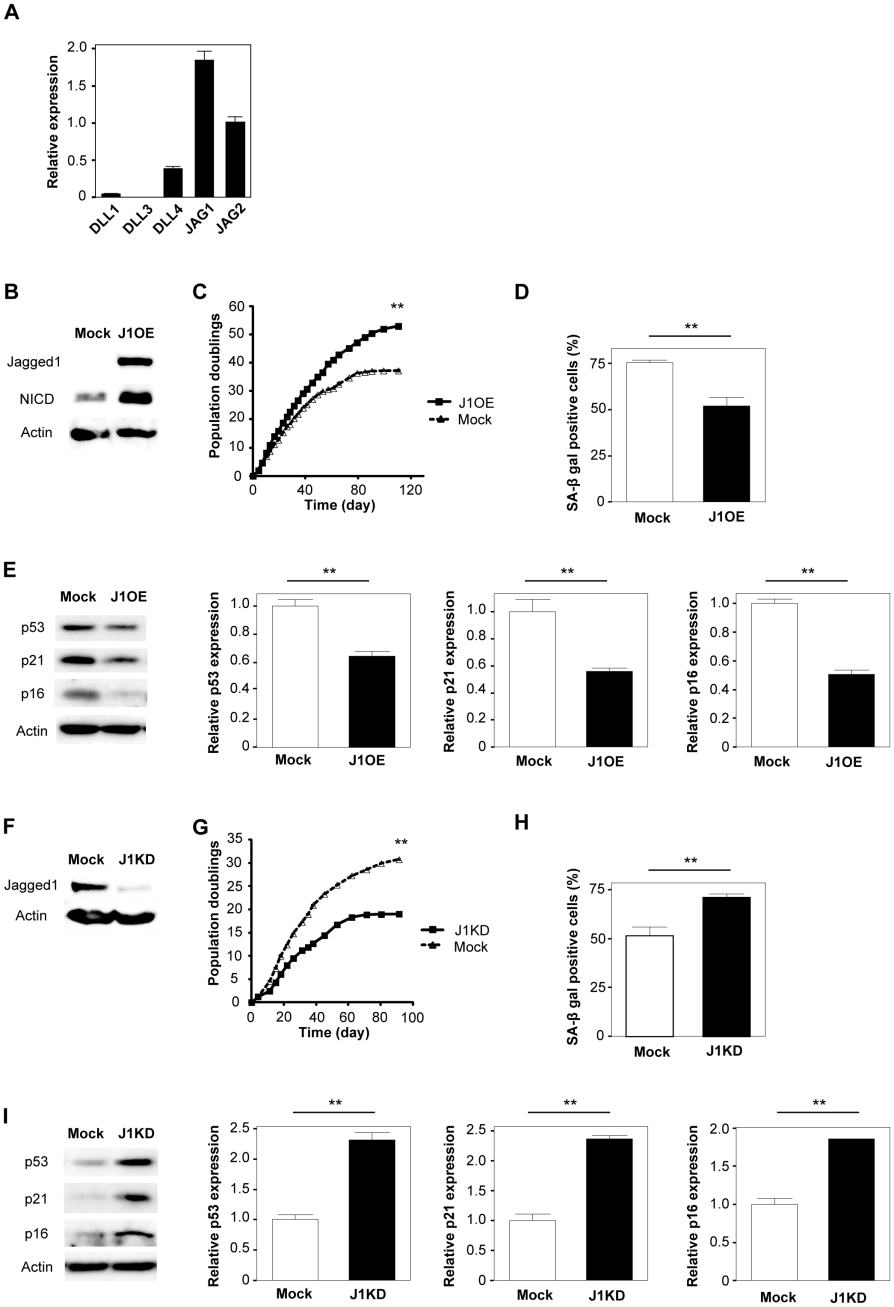
Over-expression of Jagged1 prolongs the lifespan of vascular endothelial cells. (A) Real-time PCR analysis showing the relative expression of Notch ligands (*DLL1, DLL3, DLL4, JAG1*, and *JAG2*) in human endothelial cells (n = 5). (B) Western blot analysis of Jagged1 and Notch intracellular domain (NICD) expression in human endothelial cells infected with a retroviral vector encoding Jagged1 (J1OE) or an empty vector (Mock). Actin was used as the loading control. (C) Infected cells were passaged until senescence, and the total number of population doublings was determined (n = 3). (D) Senescence-associated β-galactosidase (SA-β gal) staining of endothelial cells prepared as in Figure 2B. The graph shows quantitative data on SA-β gal-positive cells (n = 4). (E) Western blot analysis of p53, p21, and p16 expression in endothelial cells prepared as in Figure 2B. The right graphs show quantitative data on p53, p21, and p16 expression (n = 3). (F) Western blot analysis of Jagged1 in human endothelial cells infected with a retroviral vector expressing human Jagged1 shRNA (J1KD) or shControl (Mock). (G) Infected cells were passaged until senescence, and the total number of population doublings was determined (n = 3). (H) Senescence-associated β-galactosidase (SA-β gal) staining of endothelial cells prepared as in Figure 2F. The graph shows quantitative data on SA-β gal-positive cells (n = 4). (I) Western blot analysis of p53, p21, and p16 in endothelial cells prepared as in Figure 2F. The right graphs show quantitative data on p53, p21, and p16 expression (n = 3). All values represent the mean ± s.e.m. *P<0.05, **P<0.01.

Because Notch signaling is induced by a receptor-ligand interaction, we speculated that Notch ligands could also be related to vascular aging. To test this concept, we infected endothelial cells with a retroviral vector encoding the Notch ligand Jagged1, which is most highly expressed by endothelial cells among the various Notch ligands (Figure 2A). Western blot analysis revealed that over-expression of Jagged1 activated Notch signaling (Figure 2B). Similar to Notch1 over-expressing cells, up-regulation of Jagged1 extended the replicative lifespan of endothelial cells along with a decrease of SA-β-gal activity, and decreased the expression of senescence-associated molecules such as p53, p21, and p16 (Figure 2C-E). Conversely, knockdown of Jagged1 by shRNA induced premature senescence with an increase of SA-β-gal activity, increased expression of p53, p21, and p16 (Figure 2F-I). These results suggest that Notch signaling induced by receptor-ligand interactions, especially that of Notch1 with Jagged1, has a crucial role in endothelial cell senescence.

### Up-regulation of Id1 inhibits premature senescence induced by Notch1 disruption

**Figure 3:**
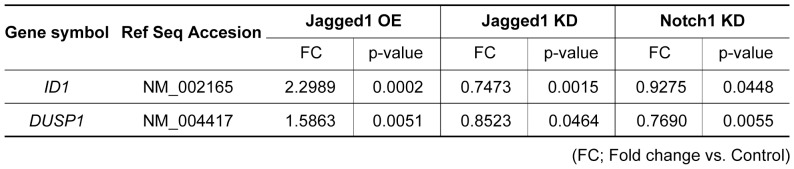
Microarray analysis in human endothelial cells. Microarray analysis of human endothelial cells showing that the Notch signaling positively regulates the expression of *ID1* and *DUSP1*. OE; over-expression, KD; knock-down.

**Figure 4:**
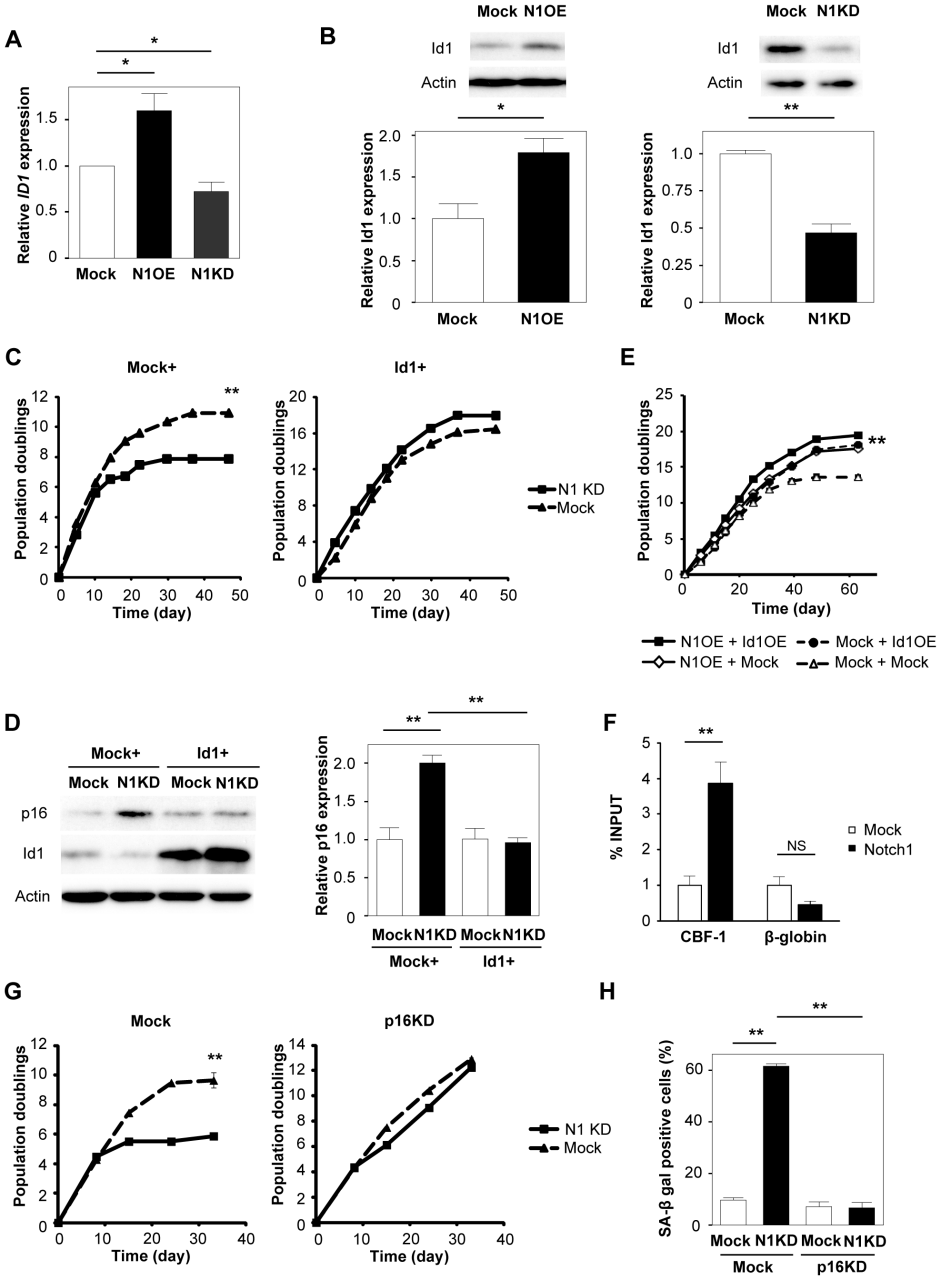
Up-regulation of Id1 inhibits premature senescence induced by Notch1 disruption. (A) Real-time PCR for the expression of *ID1* in Notch1 over-expressing endothelial cells (N1OE), Notch1 knockdown cells (N1KD), and Mock-infected cells (Mock) (n = 12). (B) Western blot analysis of Id1 in endothelial cells prepared as in Figure 4A. The graphs display quantitative data on Id1 expression (n = 3). (C) Human endothelial cells were infected with a retroviral vector encoding Notch1 shRNA (N1KD) or an empty vector (Mock). Infected cells were then transduced with pLNCX-Id1 (Id1+) or an empty vector (Mock+) and subjected to the proliferation assay as described in the legend for Figure 1B (n = 3). (D) Western blot analysis of p16 and Id1 expression in endothelial cells prepared as in Figure 4C. The right graph displays quantitative data on p16 expression (n = 3). (E) Human endothelial cells were co-infected with retroviral vectors encoding Notch1 and Id1. Infected cells were passaged until senescence, and the total number of population doublings was determined (n = 3). (F) ChIP assay of the direct association between Notch1 and Id1 in Notch1 overexpressing cells (Notch1) or mock-infected cells (Mock) (n = 5). The amount of activated Notch1 localized to the CBF1-binding element was estimated by real-time PCR. The β-globin locus was used as a negative control. (G) Human endothelial cells were infected with a lentiviral vector encoding p16 shRNA (p16KD) or an empty vector (Mock). Infected cells were then transduced with a retroviral vector encoding Notch1 shRNA (N1KD) or an empty vector (Mock) and subjected to the proliferation assay as described in the legend for Figure 1B. (H) Senescence-associated β-galactosidase (SA-β gal) staining of endothelial cells prepared as in Figure 4G. The graph displays quantitative data on SA-β gal-positive cells (n = 4). All values represent the mean ± s.e.m. *P<0.05, **P<0.01.

To further investigate the mechanism by which disruption of Notch signaling induces premature senescence of endothelial cells, we performed DNA microarray analysis and identified inhibitor of DNA binding 1 (Id1) as a potential target of the Notch signaling (Figure 3). Consistent with the results of microarray analysis, real-time PCR and western blotting showed that Id1 expression was increased in Notch1 over-expressing endothelial cells, whereas it was decreased in Notch1 knockdown cells (Figure 4A, B). Id1 is a basic helix-loop-helix (bHLH) protein that lacks a basic DNA-binding domain but is able to form heterodimers with other bHLH proteins, thereby inhibiting DNA binding and the transcriptional activity of these proteins. Since Id1 was reported to negatively regulate the expression of p16 [18], [19], we speculated that down-regulation of Id1 induced by the disruption of Notch1 led to up-regulation of p16 and premature senescence of endothelial cells. To test this hypothesis, we co-infected human endothelial cells with retroviral vectors encoding Id1 and Notch1 shRNA. Over-expression of Id1 inhibited premature senescence induced by knockdown of Notch1 and normalized p16 expression (Figure 4C, D). We also examined the effects of co-expression of Notch1 and Id1 on cell lifespan and found no additive or synergic effects (Figure 4E), suggesting that Id1 is a crucial regulator for Notch1-induced extension of endothelial cell lifespan. When Notch signaling was activated, cleaved NICD underwent translocation to the nucleus and bound to CBF1 protein. This binding of NICD facilitated displacement of transcriptional repressors from CBF1 and recruited transcriptional co-activators, thereby leading to transcription of target genes. Our *in silico* assay identified a putative CBF1-binding site in the promoter region of *ID1*. The chromatin immunoprecipitation (ChIP) assay showed that activated Notch1 has a high affinity for the CBF1-binding element in the *ID1* promoter (Figure 4F). To investigate whether Notch1 deletion induced endothelial cell senescence via a p16-dependent pathway, we co-infected human endothelial cells with the p16 shRNA and Notch1 shRNA vectors. Notch1 deletion led to premature senescence of mock-infected cells but not p16 shRNA-infected cells (Figure 4G, H), suggesting that disruption of Notch1 promotes endothelial cell senescence via an Id1/p16-dependent pathway.

### Inhibition of p38MAPK prevents induction of premature senescence by Notch1 disruption

**Figure 5:**
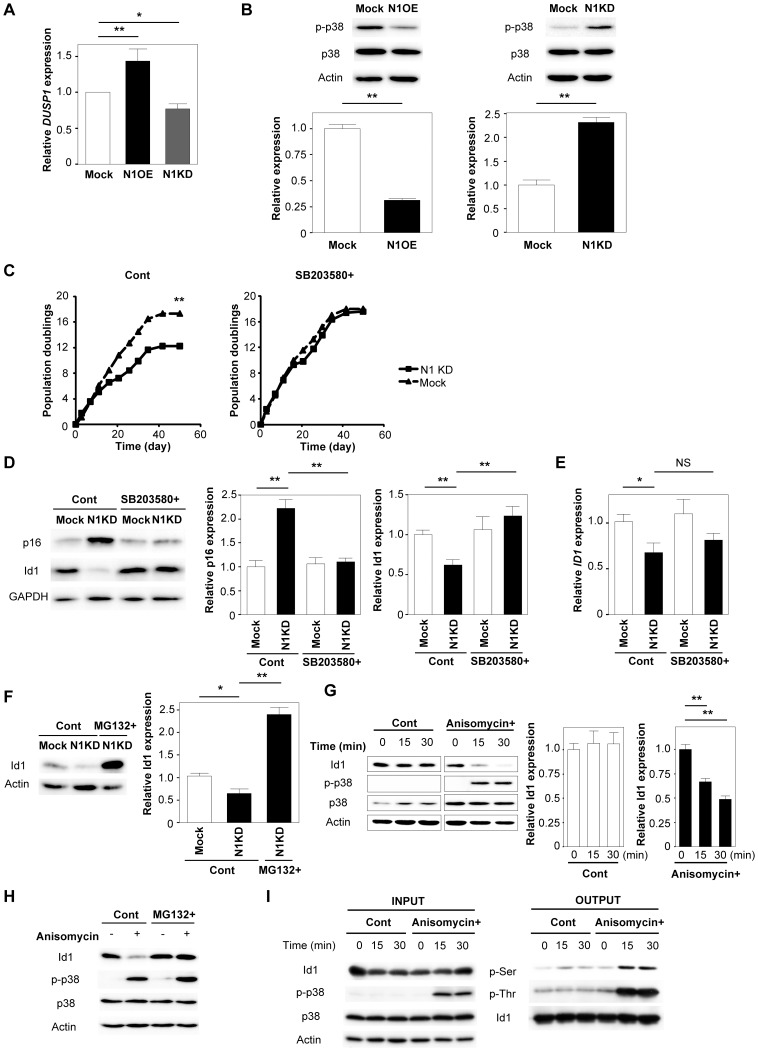
Inhibition of p38MAPK prevents induction of premature senescence by Notch1 disruption. (A) Real-time PCR for the expression of *DUSP1* (MKP1) in Notch1 over-expressing endothelial cells, Notch1 knockdown cells, and Mock-infected cells (n = 8). (B) Western blot analysis of phospho-p38MAPK (p-p38) and whole p38MAPK expression. The graphs show quantitative data on phospho-p38 expression (n = 3). (C) Human endothelial cells were infected with a retroviral vector encoding Notch1 shRNA (N1KD) or an empty vector (Mock). Infected cells were then treated with vehicle (Cont) or SB203580 (SB203580+) and subjected to the proliferation assay as described in the legend for Figure 1B (n = 3). (D) Western blot analysis of p16 and Id1 expression. The right graphs show the quantitative data on p16 and Id1 expression (n = 3). (E) Real-time PCR for the expression of *ID1* (n = 6). (F) Human endothelial cells were infected with a retroviral vector encoding Notch1 shRNA (N1KD) or an empty vector (Mock) and were treated with vehicle (Cont) or proteasome inhibitor MG132 (MG132+), after which the expression of Id1 was assessed by western blot analysis. The right graph shows quantitative data on Id1 expression (n = 3). (G) Human endothelial cells were treated with vehicle (Cont) or anisomycin (Anisomycin+) for 0, 15 or 30 minutes, after which the expression of Id1, phosphorylated p38MAPK (p-p38), and whole p38MAPK was determined by western blot analysis. The right graph shows quantitative data on Id1 expression (n = 3). (H) Western blot analysis of the expression of Id1, phosphorylated p38MAPK (p-p38), and whole p38MAPK in human endothelial cells pre-incubated with vehicle (Cont) or MG132 for 1 hour followed by treatment with or without anisomycin for 15 minutes. (I) Flag-tagged Id1 over-expressing cells were pre-incubated with MG132 for 1 hour, followed by treatment with vehicle (Cont) or anisomycin (Anisomycin+) for 0, 15, or 30 minutes. Cell lysates were immunoprecipitated with FLAG M2 agarose. Then the levels of serine phosphorylated Id1 (p-Ser), threonine phosphorylated Id1 (p-Thr), and whole Id1 were assessed by western blot analysis (right; OUTPUT). Expression of Id1, phosphorylated p38MAPK (p-p38), and whole p38 MAPK was also estimated before immunoprecipitation (left; INPUT). All values represent the mean ± s.e.m. *P<0.05, **P<0.01.

We also found that expression of MAPK phosphatase 1 (MKP1, also known as DUSP1), which inactivates p38MAPK, was associated with the Notch1 signaling, as demonstrated by DNA microarray analysis (Figure 3). Consistent with this finding, the expression of *DUSP1* was significantly up-regulated in Notch1 over-expressing cells and was down-regulated in Notch1 knockdown cells (Figure 5A). Expression of phosphorylated p38MAPK was decreased by over-expression of Notch1 and increased by knockdown of Notch1 (Figure 5B). Since MKP-1 is also reported to inactivate c-Jun N-terminal kinase (JNK) in some settings [20], [21], we examined whether MKP-1 inhibits phosphorylation of JNK and found that expression of phosphorylated JNK was unchanged by the knockdown of Notch1 or Jagged1 (Figure S3). Treatment of Notch1 knockdown cells with SB203580, an inhibitor of p38MAPK, significantly improved premature senescence induced by disruption of Notch1 along with a decrease of p16 expression (Figure 5C, D). Interestingly, although transcription of *ID1* was not altered by treatment with SB203580 (Figure 5E), expression of Id1 protein in Notch1 knockdown cells was significantly increased (Figure 5D). Expression of Id1 protein in Notch1 knockdown cells was also increased by treatment with MG132, a proteasome inhibitor (Figure 5F). Conversely, treatment with anisomycin, which activates p38MAPK, down-regulated Id1 protein expression (Figure 5G). Moreover, treatment with the proteasome inhibitor inhibited anisomycin-induced down-regulation of Id1 protein expression (Figure 5H). Activation of p38MAPK increased serine/threonine phosphorylation of Id1 protein (Figure 5I), suggesting that p38MAPK phosphorylates this protein and down-regulates Id1 expression by promoting its proteasomal degradation.

### Up-regulation of Id1 improved the phenotypic changes induced by Notch1 disruption

**Figure 6:**
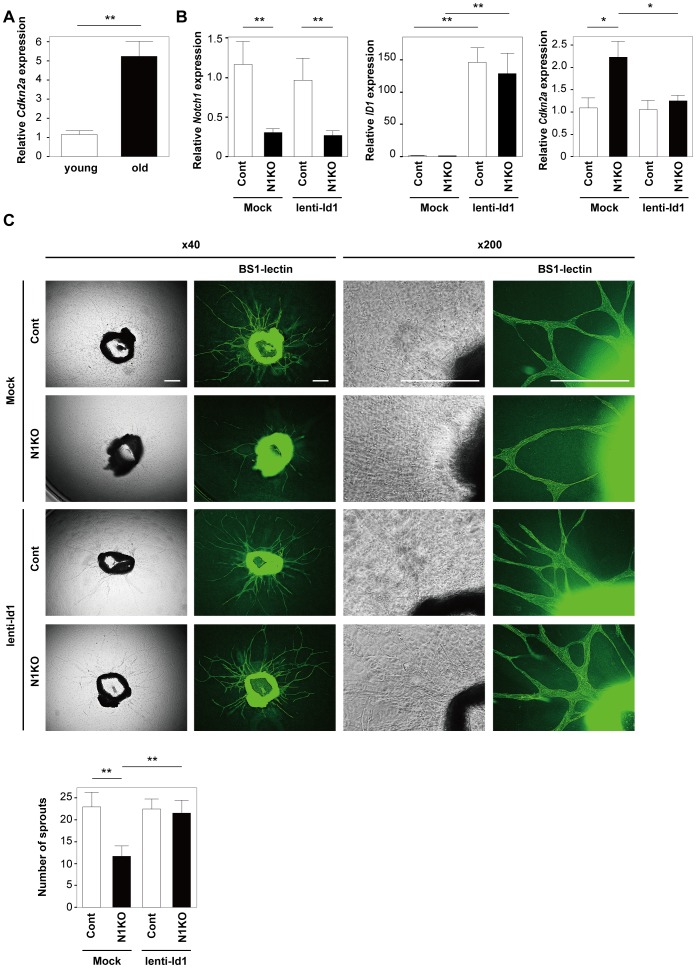
Up-regulation of Id1 improved the phenotypic changes induced by Notch1 disruption. (A) Real-time PCR analysis showing the expression of *Cdkn2a* (p16) in the aortas of young mice (12–15 weeks) or old mice (50–55 weeks) (n = 6). (B) Real-time PCR analysis showing the expression of *Notch1*, *ID1*, and *Cdkn2a* (p16) in aortas prepared as in Figure 6C (n = 6). (C) Aortic ring assay performed in endothelial cell-specific *Notch1* heterozygous knockout mice (N1KO) and their littermate controls (Cont) infected with lentivirus expressing human Id1 (lenti-Id1) or an empty vector (Mock). Cultured aortic rings were immunostained with BS1 lectin-FITC (Green). Scale bar = 100 µm. The graph displays the quantitative data for the number of sprouting cells (n = 14). All values represent the mean ± s.e.m. *P<0.05, **P<0.01.

Expression of *Cdkn2a* (the gene encoding p16 protein) in the aortas of mice was up-regulated with aging (Figure 6A). To investigate whether Notch signaling was involved in vascular aging, we performed the aortic ring assay in endothelial cell-specific *Notch1* heterozygous knockout (Tie2-cre^+^ *Notch1* ^lox/+^, (N1KO)) mice. We utilized Tie2-cre^+^ *Notch1* ^lox/+^ mice in our study because Tie2-cre^+^ *Notch1* ^lox/lox^ mice show embryonic lethality[22]. The expression of *Notch1* was significantly down-regulated in the aortas of N1KO mice (Figure 6B). Consistent with the *in vitro* data on Notch1 knockdown cells, aortic expression of *Cdkn2a* was significantly higher in N1KO mice than in littermate controls (Figure 6B). Consequently, endothelial cell proliferation was markedly impaired in *ex vivo* aortic cultures derived from N1KO mice compared with their littermate controls (Figure 6C). Introduction of Id1 with a lentiviral vector significantly increased cell proliferation in N1KO mice together with decreased expression of *Cdkn2a* (Figure 6B, C), indicating that Notch signaling positively regulates the lifespan of endothelial cells via the down-regulation of p16.

## Discussion

In the present study, we demonstrated that the Notch signaling pathway is crucially involved in the process of vascular aging. Down-regulation of Notch signaling reduced Id1 and MKP1 expression and also accelerated endothelial cell senescence via a p16-dependent pathway. It has been reported that down-regulation of Notch signaling is related to various age-associated conditions. A recent study revealed that Notch signaling is down-regulated in aged skeletal muscle, and a decline of Notch activity was shown to impair the proliferation of muscle precursor cells and their production of myoblasts for muscle regeneration [9], [10].

The role of Notch signaling in endothelial cell proliferation has been controversial. A study by Venkatesh et al. showed that up-regulation of Notch signaling by NICD inhibits the proliferation of endothelial cells [23]. In line with our results, Notch activation was reported to down-regulate expression of p21 [24]. It is well accepted that Notch signaling plays a crucial role in the development of various malignancies [25]–[27]. Activating Notch mutations have been reported in human leukemia and breast cancer. It also has been reported that oncogenic stimuli provoke premature senescence in a variety of human somatic cells [28]. For example, constitutive activation of Ras or Akt has been shown to cause premature senescence [29], [30], and both of these signaling molecules are known to promote cell proliferation and contribute to tumorigenesis. In this regard, cellular senescence is thought to be a defensive mechanism against malignant transformation. We found that introduction of NICD led to premature senescence of human endothelial cells along with up-regulation of negative regulators of the cell cycle including p16, whereas introduction of Notch1 prolonged the lifespan of endothelial cells. Thus, constitutive activation of the Notch signaling pathway with NICD could act as an oncogenic stimulus that leads to premature senescence, whereas activation of this pathway at a physiological level by full length Notch1 may result in extension of the cellular lifespan. Although activation of Notch signaling was reported to reduce telomerase activity [31], we did not find any differences of telomere length or telomerase expression between Notch1-infected and mock-infected cells. Because it is well-known that mice have high telomerase activity and long telomeres [32], [33], it is unlikely that Notch signaling regulates endothelial cell proliferation by modulating telomerase activity in mice.

Tip cells are non-proliferative highly motile cells that are restricted to the tips of sprouts [34]. In contrast, stalk cells are highly proliferative and form the trunks of new blood vessels [34]. In vascular sprouts, the tip cells express a high level of delta-like (Dll) 4 and a low level of Notch1, while the stalk cells show high expression of both Notch1 and Jagged1 [35]. There have been previous reports demonstrating that inhibition of Dll4-mediated Notch signaling leads to an increase in the number of filopodia and sprouting tips, and that Jagged1 antagonizes the effects of Dll4 on sprouting angiogenesis [36]–[38]. The genetic models used in these studies included endothelial-specific Notch1 homozygous knockout mice (an inducible model) and Dll4 heterozygous knockout mice. We utilized endothelial cell-specific Notch1 heterozygous knockout (Tie2-cre^+^ *Notch1* ^lox/+^) mice and obtained the opposite results. Consistent with our findings, however, it has been reported that endothelial cell-specific or systemic Notch1 heterozygous knockout results in impairment of postnatal angiogenesis [39], [40]. Our *in vitro* experiments clearly demonstrated that activation of the Jagged1/Notch1 pathway promotes endothelial cell proliferation. Collectively, these results suggest that strong inhibition of the Notch1 signaling promotes vascular sprouting by attenuating Dll4-dependent signaling in the tip cells, while moderate inhibition of this pathway leads to significant reduction of Jagged1-dependent signaling in the stalk cells that results in impaired angiogenesis, but does not affect Dll4-dependent signaling in the tip cells.

We also found that the Notch pathway positively regulates Id1 and MKP1 expression. Some evidence has been published suggesting a potential association between Notch and Id1 [41], [42], and our results indicate that *ID1* is a target gene of Notch1. In agreement with the results of our microarray analysis, Kondoh et al. showed that Notch signaling suppresses p38MAPK activity via induction of MKP1 during myogenesis [43]. We further demonstrated that Notch1-induced up-regulation of MKP1 stabilized Id1 protein by inhibiting p38MAPK-induced degradation, leading to prolongation of the endothelial cell lifespan. Taken together, our results suggest that activation of Notch1 could be a new therapeutic target for treating age-associated vascular diseases.

## Supporting Information

Figure S1 **The effects of NICD overexpression.** (A) Western blot analysis for the expression of Notch intracellular domain (NICD) and p16 in endothelial cells infected with Notch1 (N1OE), NICD, or an empty vector (Mock). (B) Population doublings of endothelial cells infected with Notch1 (N1OE), NICD, or an empty vector (Mock) (n = 3). **P<0.01 vs. Mock. (C) Real-time PCR analysis showing the expression of p53 (*TP53*), p21 (*CDKN1A*), and p16 (*CDKN2A*) in cells as prepared in Figure S1B (n = 5–9). The results of N1OE and Mock are also shown in Figure 1A. Data are shown as the mean ± s.e.m. *P<0.05, **P<0.01.
(TIF)

Figure S2 **The effect of Notch1 overexpression on endothelial cell senescence is independent of telomere shortening.** (A) Real-time PCR for the relative expression of telomerase reverse transcriptase (TERT) in endothelial cells infected with Notch1 (N1OE) or an empty vector (Mock) (n = 6). PC3 (PC3) is a human prostate cancer cell line that was used for a positive control. (B) Relative telomere length in endothelial cells infected with Notch1 (N1OE) or an empty vector (Mock) (n = 4). All data are shown as the mean ± s.e.m. *P<0.05, **P<0.01.
(TIF)

Figure S3 **The expression of phosphorylated JNK of Notch1 or Jagged1 knock-down cells.** Western blot analysis of phospho-JNK (p-JNK) and whole JNK expression in Notch1 (N1KD) or Jagged1 (J1KD) knock-down cells. The graphs indicate the quantification relative to whole JNK (n = 7). Values are the mean ± s.e.m.
(TIF)
