# Supplementary figures and images for: Notch Signaling Regulates the Lifespan of Vascular Endothelial Cells via a p16-Dependent Pathway
Source: PLoS One. 2014 Jun 20;9(6):e100359. doi: 10.1371/journal.pone.0100359 (PMC4065107; doi:10.1371/journal.pone.0100359)

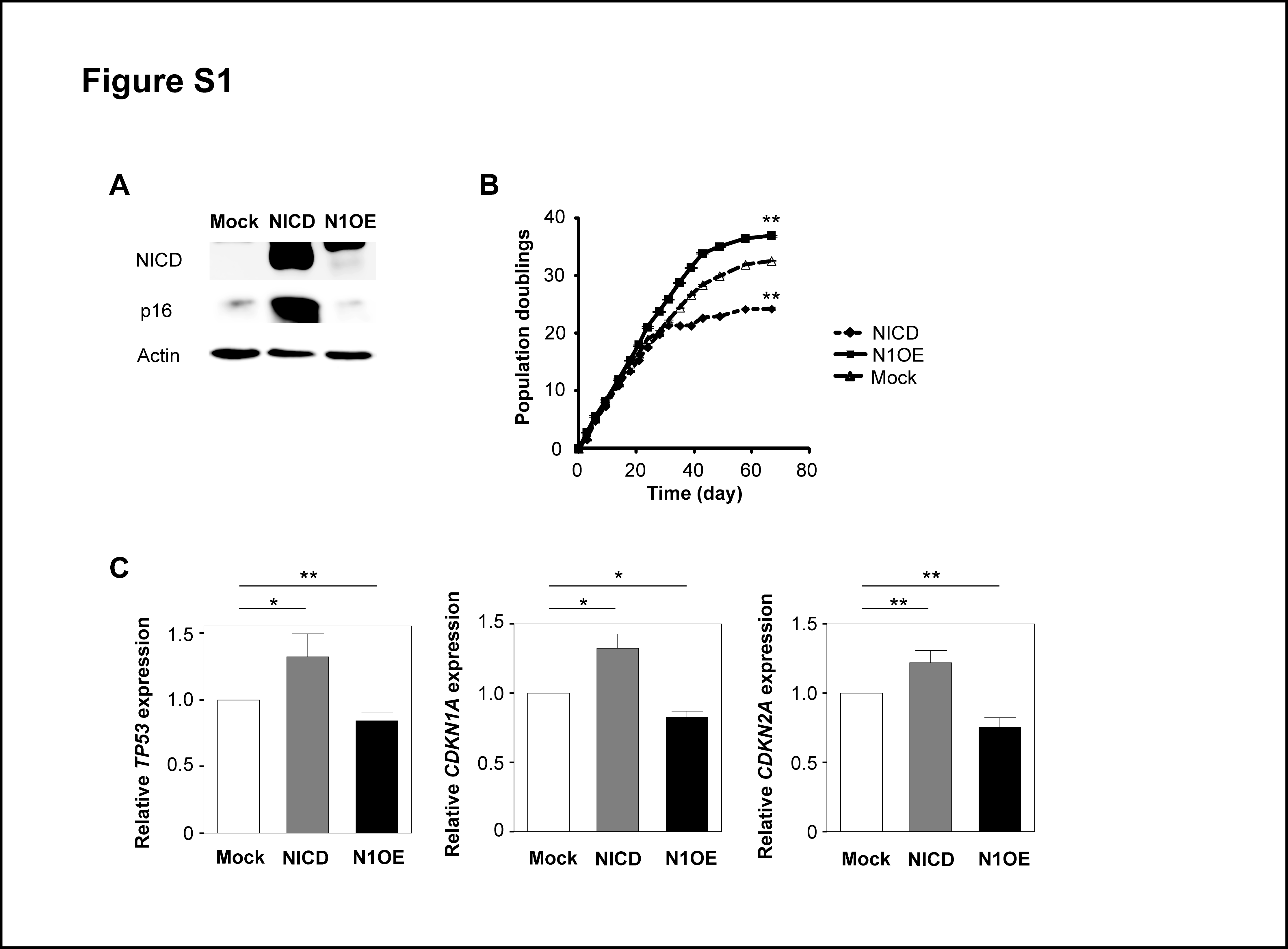

Supplement: Figure S1 — The effects of NICD overexpression. (A) Western blot analysis for the expression of Notch intracellular domain (NICD) and p16 in endothelial cells infected with Notch1 (N1OE), NICD, or an empty vector (Mock). (B) Population doublings of endothelial cells infected with Notch1 (N1OE), NICD, or an empty vector (Mock) (n = 3). **P<0.01 vs. Mock. (C) Real-time PCR analysis showing the expression of p53 (TP53), p21 (CDKN1A), and p16 (CDKN2A) in cells as prepared in Figure S1B (n = 5–9). The results of N1OE and Mock are also shown in Figure 1A. Data are shown as the mean ± s.e.m. *P<0.05, **P<0.01. (TIF) [file pone.0100359.s001.tif]

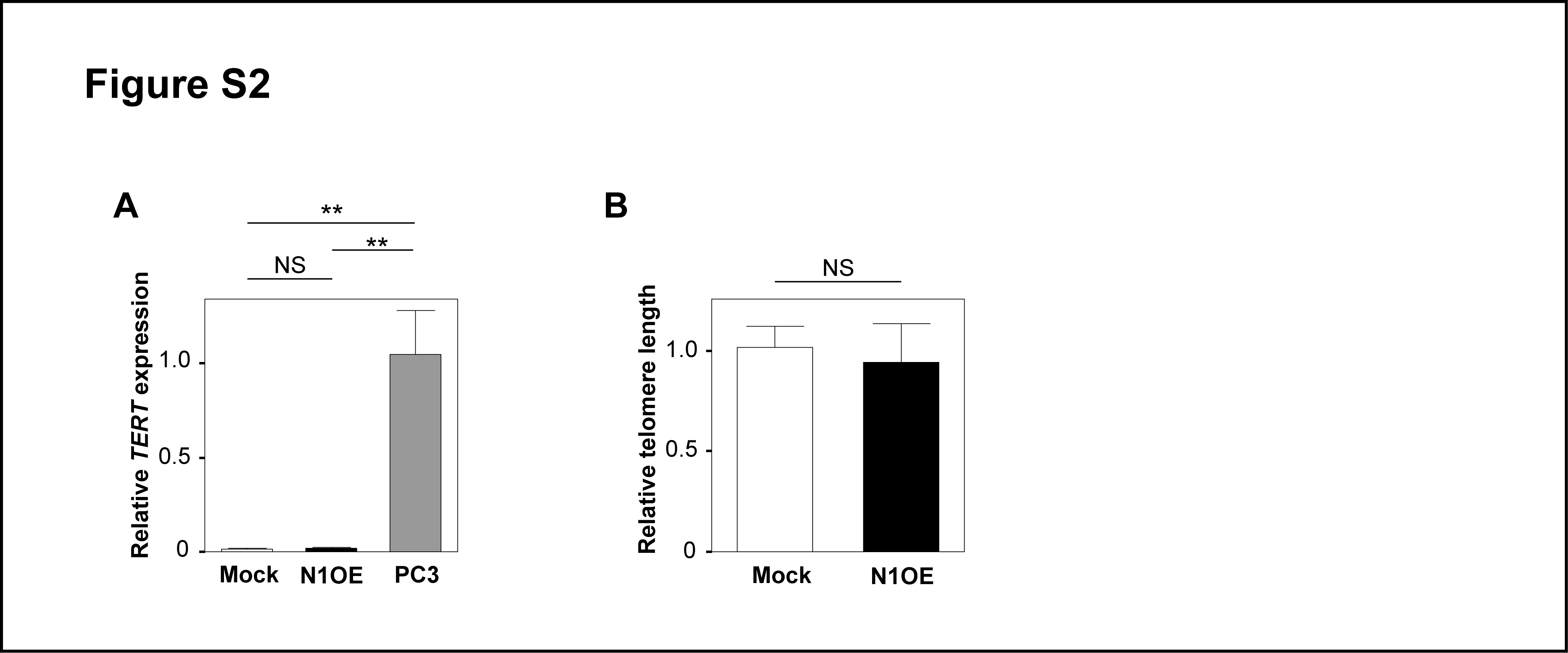

Supplement: Figure S2 — The effect of Notch1 overexpression on endothelial cell senescence is independent of telomere shortening. (A) Real-time PCR for the relative expression of telomerase reverse transcriptase (TERT) in endothelial cells infected with Notch1 (N1OE) or an empty vector (Mock) (n = 6). PC3 (PC3) is a human prostate cancer cell line that was used for a positive control. (B) Relative telomere length in endothelial cells infected with Notch1 (N1OE) or an empty vector (Mock) (n = 4). All data are shown as the mean ± s.e.m. *P<0.05, **P<0.01. (TIF) [file pone.0100359.s002.tif]

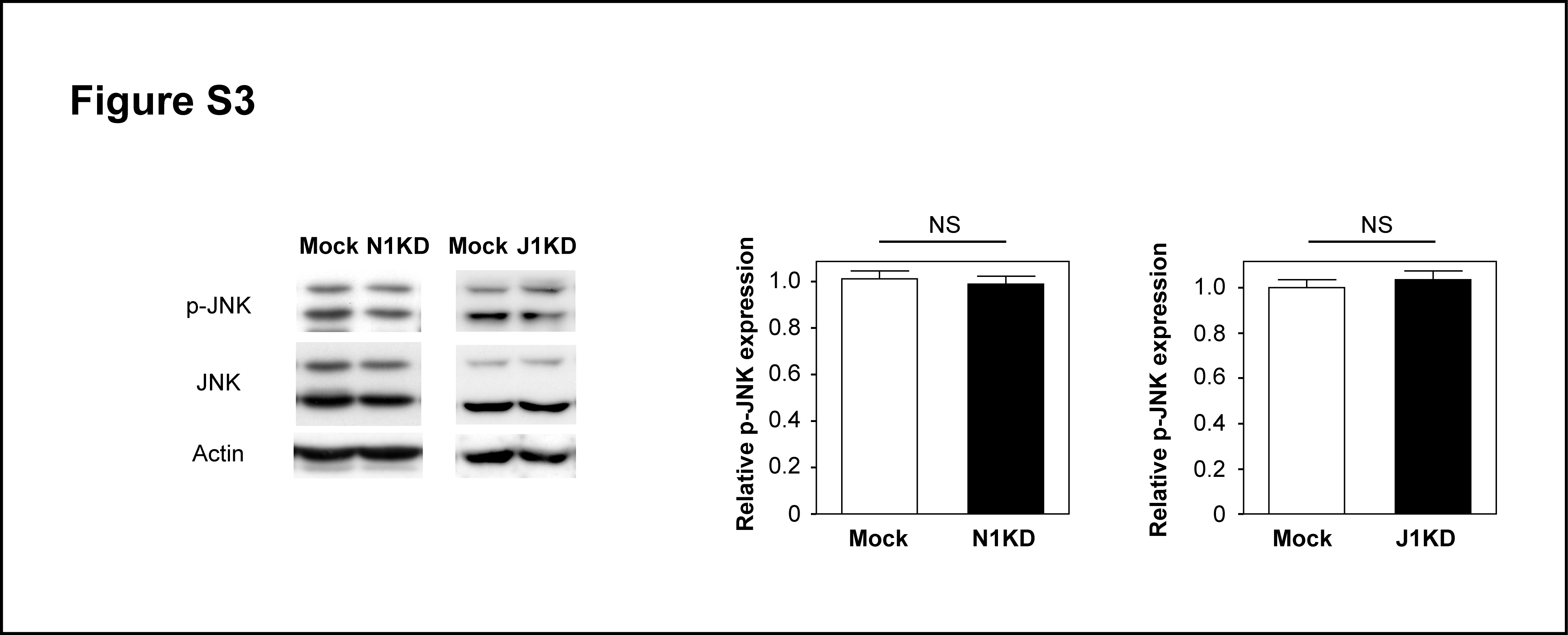

Supplement: Figure S3 — The expression of phosphorylated JNK of Notch1 or Jagged1 knock-down cells. Western blot analysis of phospho-JNK (p-JNK) and whole JNK expression in Notch1 (N1KD) or Jagged1 (J1KD) knock-down cells. The graphs indicate the quantification relative to whole JNK (n = 7). Values are the mean ± s.e.m. (TIF) [file pone.0100359.s003.tif]
